# Aligning to a New Normal During COVID-19

**DOI:** 10.1093/geroni/igab046.1087

**Published:** 2021-12-17

**Authors:** Lizette Munoz

**Affiliations:** Mount Sinai Health System, New York, New York, United States

## Abstract

The Acute Life interventions Goals and Needs Program (ALIGN) is an inter-professional team of medical and social work providers dedicated to offering time-limited intensive ambulatory care to the most complex, high cost, high needs older patient population at Mount Sinai Hospital in NYC. During the 2020 COVID19 pandemic, ALIGN pivoted to focus on emergency planning actions. Such actions included language and culturally concordant goals of care discussions with patients and family, completion of electronic Medical Orders for Life Sustaining Treatment, reassessment of patient’s social determinants of health, determination of adequate access to food, medication, and emotional support to those alone and isolated, and assistance with video telemedicine. ALIGN’s model of care has shown how adaptable this program and others were during the height of the pandemic.

